# Schistosome Egg Migration: Mechanisms, Pathogenesis and Host Immune Responses

**DOI:** 10.3389/fimmu.2018.03042

**Published:** 2018-12-20

**Authors:** Alice H. Costain, Andrew S. MacDonald, Hermelijn H. Smits

**Affiliations:** ^1^Department of Parasitology, Leiden University Medical Center, Leiden, Netherlands; ^2^Lydia Becker Institute of Immunology and Inflammation, University of Manchester, Manchester, United Kingdom

**Keywords:** *Schistosoma mansoni*, intestine, endothelium, type 2 immunity, immune modulation

## Abstract

Many parasitic worms possess complex and intriguing life cycles, and schistosomes are no exception. To exit the human body and progress to their successive snail host, *Schistosoma mansoni* eggs must migrate from the mesenteric vessels, across the intestinal wall and into the feces. This process is complex and not always successful. A vast proportion of eggs fail to leave their definite host, instead becoming lodged within intestinal or hepatic tissue, where they can evoke potentially life-threatening pathology. Thus, to maximize the likelihood of successful egg passage whilst minimizing host pathology, intriguing egg exit strategies have evolved. Notably, schistosomes actively exert counter-inflammatory influences on the host immune system, discreetly compromise endothelial and epithelial barriers, and modulate granuloma formation around transiting eggs, which is instrumental to their migration. In this review, we discuss new developments in our understanding of schistosome egg migration, with an emphasis on *S. mansoni* and the intestine, and outline the host-parasite interactions that are thought to make this process possible. In addition, we explore the potential immune implications of egg penetration and discuss the long-term consequences for the host of unsuccessful egg transit, such as fibrosis, co-infection and cancer development.

## Introduction

Schistosomiasis is a chronic and potentially lethal tropical disease, mainly caused by the parasitic blood flukes *Schistosoma mansoni, S. haematobium, and S. japonicum*. Schistosomes have evolved to develop and thrive in their infected hosts, with untreated infections generally persisting for 3–10 years and a minority of infected individuals developing severe, life-threatening pathology (1). Common among parasites, schistosomes possess rather peculiar life cycles. This includes stages within definitive human hosts and secondary snail vectors, transformation through various larval forms, and—importantly—a unique process of egg migration to leave their human host. In this essential life cycle event, schistosome eggs pass from the host vasculature, across intervening tissue and into the environment via host excretions. This enigmatic process, and its pathological/immunological consequences, is the focus of this review, with particular emphasis placed on the intestinal response to *S. mansoni*.

Adult *S. mansoni* worms reside deep within the mesenteric veins of the intestine, where they feed on blood and acquire nutrients necessary for growth, development, and egg production (2). Each worm pair produces ~300 eggs daily, which exit the host by moving from the depths of the mesenteric vessels, across the intestinal wall and into the intestine lumen (3, 4). Importantly, as schistosome eggs are not in possession of any obvious motility mechanisms themselves, their expulsion is likely to be heavily reliant on host-driven processes. However, successful egg passage is not guaranteed. Approximately half of all deposited eggs never reach the intestine, but instead are swept to the liver, where they evoke strong granulomatous inflammation, as characterized by the infiltration of alternatively activated (AA) macrophages, eosinophils and T-helper 2 (Th2) cells, with additional fibroblast proliferation and generation of extracellular matrix (3–5). For the remainder of intestinally-bound eggs, success is still not certain. Firstly, eggs remain viable for a mere 2–3 weeks following oviposition, providing them with a relatively short timeframe to make this journey (6, 7). Secondly, due their high antigenicity and continual release of antigens and other metabolites, transiting eggs are easily detected by the host immune system, becoming the focal point of inflammatory granulomatous reactions. If these responses are too extreme, a variety of immune-pathologic sequelae will follow (8).

**Figure 1:**
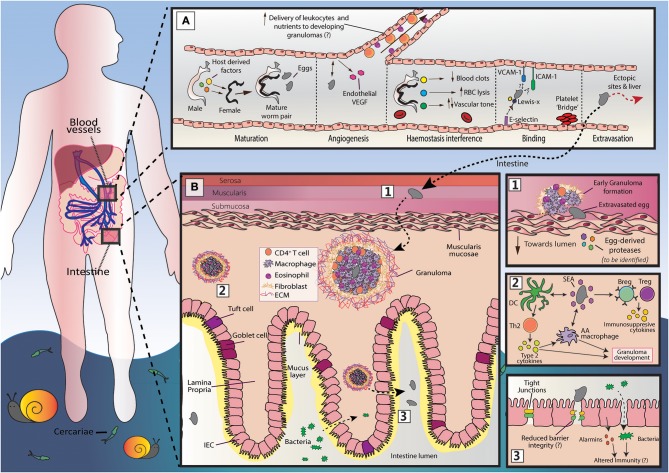
An overview of *S. mansoni* egg migration. Schistosome egg transit is facilitated by a series of host interactions at the intestinal and vascular interface. **(A)** The development of schistosomes into sexually mature, egg-producing adults occurs within the portal vein (~3–5 weeks post infection) and requires the transduction of host-derived signals (including those from the innate and adaptive immune system) to the developing worm pair. Once sexual maturity is reached, worm pairs migrate toward the mesenteric vessels, where the females lay approximately 300 eggs per day and actively modulates the intravascular environment to support their long-term survival. The production of eggs at ~5–6 weeks post infection is a milestone event in the schistosome life cycle, that is characterized by induction of a marked Th2 response and angiogenesis. Notably, the generation of a Th2 response by the host is critical for egg passage, and new vessel formation may favor egg transit, promoting the recruitment of immune cells and nutrients to developing granulomas. Freshly deposited eggs cannot move by themselves and must somehow attach and extravasate the endothelium. Although yet to be fully defined, this process may involve E-selectin:-Lewis-x interactions, and participation from platelets, ICAM-1 and VCAM-1. While a large proportion of eggs successfully penetrate the endothelium and reach intestinal tissue, many are swept to the liver or other distal locations (e.g., brain or spinal cord). Since schistosome eggs are unable to transit through these organs, overwhelming tissue pathology and inflammation may ensue. **(B)** Once schistosome eggs have passed across the host endothelium and out of the vasculature, they must cross the multi-layered intestinal wall. The host immune system responds to transiting eggs via an inflammatory granuloma response, in which individual eggs are encapsulated by immune cells [including alternatively activated (AA) macrophages, Th2 cells and eosinophils] and extracellular matrix (ECM), which protects host tissues from egg-derived toxins, but ultimately leads to formation of fibrotic lesions. For unknown reasons, granulomatous responses need to successfully develop for effective egg excretion from the host. Accordingly, schistosomes and their host have co-evolved a wide range of mechanisms to skew the host immune response toward granuloma-inducing Th2 profile. These include the ability of soluble egg antigens (SEA) to promote alternative activation in macrophages and to condition dendritic cells (DCs) for Th2 polarization. However, to prevent unwanted bystander tissue damage and potentially fatal immunopathology, schistosomes also implement various strategies to dampen host immunity and expanded regulatory networks (Bregs and Tregs). There remain many unknowns surrounding egg migration. This includes the molecules secreted by eggs to disrupt host barriers and modulate immune responses and, importantly, how egg penetration and intestinal ‘leakiness' may influence local and systemic immune reactions.

As our understanding of schistosome immunobiology has increased, it has become increasingly obvious that schistosomes implement a variety of strategies to ensure efficient egg transit. Within the vasculature, egg extravasation is promoted by angiogenesis, endothelial activation, and interactions with blood clotting components (9, 10). In the intestinal tissues, schistosomes exert a variety of immunomodulatory influences to support granuloma formation around transiting eggs, which is an essential process in egg excretion (11–14). Directly related to this, and to prevent overwhelming immunopathology, schistosomes guide the immune response toward a more regulatory phenotype during chronic disease. In this review we discuss the strategies employed by schistosomes to favor egg passage, and outline the potential immune implications and pathological consequences that may follow (Figure 1).

## Endothelial extravasation

### Maturation and Mesenteric Migration

Before egg production can commence, schistosomes need to mature to adulthood while navigating from the skin, via the lungs to the mesenteric veins of the intestine (*S. mansoni* and *S. japonicum*, causing hepatosplenic disease) or bladder (*S. haematobium*, causing urinary schistosomiasis) (15–17). Worm maturation occurs in the blood vessels and requires the transduction of host-derived signals from male worms to their female partner (18). Signals from the adaptive and innate immune system are thought to be intimately linked with this process (18). Notably, worm growth and reproductive activity is severely stunted in the absence of CD4+ T cells, but can be sufficiently restored through repeated stimulation of innate immunity via toll-like receptor signaling or inflammasome activation by endogenous danger signals (18–21). The specific immunological factors that guide parasite development remain poorly defined and controversial. For instance, while a functional role for interleukin (IL)-7 in parasite development is generally agreed upon, there is ongoing controversy surrounding the role of TNF, with studies showing that both TNF neutralization and administration can promote egg production (21–23).

At approximately 4–6 weeks post infection, sexually mature worm pairs move to the mesenteric vessels, which is the site of oviposition (17). Although eggs can be found throughout the vasculature, certain sites may be favored (15). In mice, oviposition appears to be concentrated in the Peyer's patches of the small intestine, while in primates and man, eggs are more readily detected in the colon and rectum (15, 16, 24, 25). Why oviposition shows such patterns is subject to speculation. It is possible that worm migration is dictated by host-derived signals (e.g., hormones or digestion products absorbed across the intestine wall) or that worms preferentially exploit regions of low sheer stress and high vascularization to avoid eggs being swept away with the blood stream (15). Alternatively, blood vessel diameter could be the major determinant, with adult worm pairs being relatively large (~0.5 mm in diameter and up to 10 mm long) in comparison to the mesenteric vessels that they reside in Da'dara et al. (26). Furthermore, while *S. mansoni* eggs are laid diffusely across the intestine and seldom produce bulky, concentrated lesions, *S. haematobium* and *S. japonicum* worms tend to deposit eggs in a few areas where a large number of them are concentrated (15, 16). These deposition patterns may reflect the unavoidable clumping of worms within host vessels or, alternatively, worms may be attracted to factors at the site of eggs-induced lesions, including substances released from breached blood vessels (15). Finally, it is possible that egg aggregation supports extravasation, with the build-up of egg-derived proteases creating channels from the intravascular to intraluminal space (27).

Even though migrating worm pairs are clear potential obstructions to blood flow, schistosome infections are not associated with enhanced blood clotting. In fact, individuals with advanced hepatosplenic schistosomiasis have a reduced level of blood coagulation factors, and blood clots are not observed around worms in host vessels (28–31). Experimental studies have also shown schistosome infections to impact blood-coagulation, where blood from 7-week infected mice coagulates more rapidly than control, with faster lysis of the clot formed (26). However, *ex vivo* studies involving the exposure of adult worms to blood from infected or non-infected mice, demonstrate an anticoagulant effect of the adult parasite (26). Mechanistically, there is strong evidence indicating that that schistosomes directly modulate the host haemostatic system via a variety of bioactive secretory products and molecules on the schistosome's outer-surface (tegument) (9). For instance, schistosomes inhibit blood clot formation and/or promote blood clot lysis through the activities of several tegumental enzymes, including enolase, SmSP2, SmAP and SmCalp1&2, and vascular tone is modified through the activities of SmSP2 and SmPOP (32–36). Altogether, such processes can be viewed as a schistosome survival mechanism in the blood stream, likely promoting residence and movement while preventing unwanted vessel occlusion.

### Endothelial Adherence

Schistosome eggs are striking structures, encased by a rigid network of cross-linked proteins and, in the case of *S. mansoni*, characterized by a large protruding lateral spine. Due to the high rigidity and inflexibility of their outer shell, schistosome eggs must rely on external forces to bring them toward to the endothelial lining (37). It has long been suggested that the active migration of endothelial cells over schistosome eggs, brings the eggs into close contact with the vessel lining (38). More recently, video imaging has suggested that female worms prompt egg-endothelial associations via strong muscular contractions at their genital pore (dorsiflexion) that thrusts their eggs into the endothelium (7). Once brought into close contact with the endothelium, eggs likely tether themselves to endothelial surface adhesion molecules, including ICAM-1, VCAM-1 and E-selectin (39, 40). However, while binding to E-selectin is likely mediated by egg-shell components glycosylated with Lewis-x motifs (40), there are no obvious integrin-like structures within the egg-shell identified yet that bind ICAM-1 or VCAM-1. Interestingly, ICAM-1 shows strong upregulation in response to eggs and SEA (39), and soluble ICAM-1 levels are constitutively higher in schistosome-infected individuals and positively correlate with egg excretion rates (41). Additionally, there is evidence to show that ICAM-1 not only mediates egg binding, but also participates in the generation of granulomatous inflammation around parasite eggs, by regulating leukocyte trafficking, vascular permeability and modulating T cell responsiveness to soluble egg antigens (SEA) (39, 41, 42). As later discussed, intact granuloma formation is essential for successful egg expulsion.

Freshly deposited eggs are immediately surrounded by cells and proteins of the haemostatic system, including the plasma proteins von Willebrand factor (VWF), fibrin and fibrinogen (7, 43, 44). In addition to ICAM-1, E-selectin and VCAM-1, schistosome eggs may bind to these haemostatic components to promote their anchorage to the endothelium and to prevent them from being swept away with circulation. Indeed, the administration of platelet inactivating drugs to *S.mansoni*-infected mice results in significantly diminished egg excretion rates (45). By closely analysing the interactions between eggs and such components, deWalick and colleagues demonstrated that the schistosome egg-shell directly binds to VWF: an adhesive glycoprotein that tethers clotting material (such as platelets) to the activated endothelium (43, 46). This binding is suggested to benefit egg extravasation in two ways. VWF could form a direct bridge between eggs and the extracellular matrix, and/or the binding of VWF to clotting material may induce stable clot formation, making it easier for eggs to adhere to the endothelium.

The role of the schistosome spine in egg migration is not known. Given that *S. japiconium* eggs are virtually spineless, it is unlikely that the schistosome spine plays a major function. In fact, a recent report comparing egg morphology between Praziquantel resistant and susceptible *S. mansoni* infection suggests that the spine actually hinders egg transit (47). More specifically, resistant strain eggs were shown to have smaller lateral spines than susceptible strain eggs, and were also more frequently shed into host feces (47). Thus, from an evolutionary standpoint, perhaps *S. japiconium* has taken advantage of spine absence. However, based on the repeated observation that *S.mansoni* eggs clump to one another at their spine tip, it is also possible that spine-to-spine clumping enhances the cytotoxicity of freshly deposited eggs, promoting channel formation through host tissues and/or accelerating granuloma development (27, 48).

### Eggs and Angiogenesis

Schistosomes not only reside and produce eggs within blood vessels, but also appear to promote their formation (49). Angiogenesis is a complex process in which new vessels develop from pre-existing ones, creating an environment that favors tissue growth and repair (50). This sequential process is guided by pro-angiogenic factors (such as VEGF, angiopoietin and inflammatory cell-derived chemokines) which instruct endothelial cell activation, proliferation and reorganization (50). Evidence for schistosome-induced angiogenesis can be found in both human studies and experimental models. During murine infection, vascularity is significantly enhanced in areas of high egg concentration (including the Peyer's patches) and, when angiogenesis is inhibited, there is a reduction in worm load and hepatic egg deposition (10, 24, 51). In human studies, mucosal biopsies containing *S. haematobium* eggs are more vascularized than healthy, egg-free control tissue (52). In addition, schistosomiasis patients have significantly higher serum VEGF levels than healthy individuals, or those with active hookworm infections (10) Schistosomes likely promote neovascularization to sustain their life cycle, for several reasons. First, the remodeling of intestinal vasculature may increase the number of worms the blood vessels can accommodate and reduce egg “spill over” into hepatic tissue (24). Second, angiogenic responses could enable the recruitment of leukocytes to developing granulomas, and ensure an adequate supply of oxygen and nutrients at these sites (53). Third, increased vessel density may impair intestinal tissue, making it easier for eggs to disrupt and move through (52). Finally, similar to what has been observed in cancer, growth of new vessels would maintain blood flow in scenarios of vessel occlusion (e.g., by worm pairs and their eggs). Furthermore, conditions created by vessel occlusion such as hypoxia, acidic PH and low glucose concentration, may also contribute to the neovascularization observed (54).

While adult worms have poorly defined roles in the induction of angiogenesis, secretory products of schistosome eggs (soluble egg antigens, or SEA) have been shown to instruct angiogenesis via direct and indirect mechanisms. Investigations using human umbilical vein endothelial cells have shown that SEA can directly encourage endothelial cell proliferation, migration, sprouting and production of VEGF (54, 55). The extent of angiogenic activity can be influenced by host genetics, and lies within the glycoprotein fraction of SEA (56, 57). Indirectly, SEA induces angiogenesis through the actions of alternatively activated (AA) macrophages and hedgehog signaling (58). In this case, SEA stimulates macrophage secretion of biologically active hedgehog ligands, which subsequently activate hedgehog-responsive endothelial cells to proliferate and secrete angiogenic factors. The fact that sprouting blood vessels are more frequently observed around viable ova as opposed to dead or dying calcified ova, strongly suggests that substances actively secreted from eggs are responsible for new blood vessel formation (52).

In addition to angiogenesis, endothelial activation by schistosomes may also support granuloma formation. Schistosome triggered VEGF significantly increases proliferation of and extracellular matrix deposition by hepatic stellate cells, which are the main source of extracellular matrix around schistosome eggs in the liver (59). Whether similar mechanisms are involved in intestinal granuloma formation is unknown, though it is tempting to speculate that endothelial cells lining mesenteric vessels could be activated upon worm encounter to secrete pro-fibrotic factors, such as VEGF, IL-13, TGF-β, or IL-33, to intestinal-resident cells involved in fibrosis. IL-33, for instance, is constitutively expressed by endothelial cells, and has recently been shown to support liver granuloma pathology through the induction of pro-fibrotic AA macrophages (60, 61). In addition, VEGF partially regulates Th2 inflammatory responses in the lung and liver in response to schistosome eggs (62). Given that type 2 immune responses are essential for adequate granuloma formation, it would be interesting to define a role for VEGF in intestinal granulomatous reactions.

Overall, schistosomes employ a variety of strategies within host blood vessels that favor their survival and life cycle propagation. Increased understanding of these interactions could identify new targets for the prevention of severe disease during schistosomiasis. For instance, direct targeting of adult worms could prevent or reduce the production of eggs and their accumulation within host tissues, which is the primary cause of pathogenesis during schistosome infection.

## Intestinal Passage

**Figure 2:**
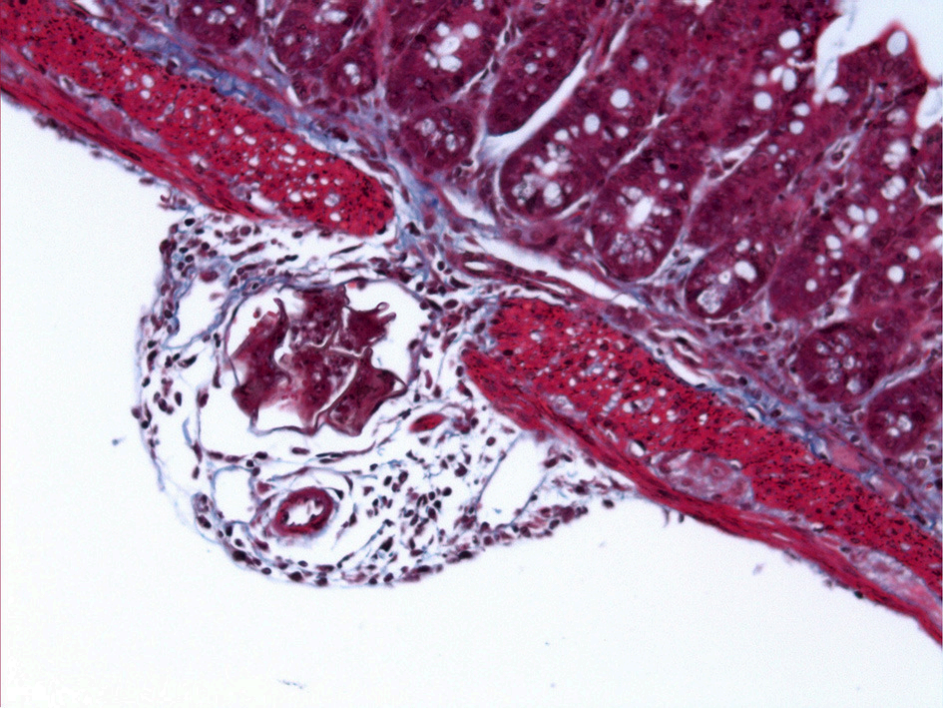
*S. mansoni* egg penetrating through the intestinal wall. Light microscope image of Masson's Trichrome stained ileal section (5 μm) from a C57BL/6 mouse 6 weeks after percutaneous infection with approximately 40 *S. mansoni* cercariae.

After successfully extravasating the mesenteric vessels schistosome eggs are confronted with a much larger anatomical hurdle: the intestinal wall. This extensive barricade is the host's largest interface with the external environment, its luminal side in constant exposure to the contents of the intestines, including innocuous (food) antigens, commensals and pathogens (63). To efficiently segregate the host from this hazardous environment, the intestinal wall incorporates a dense network of immune cells and an innermost monolayer of tightly aligned intestinal epithelial cells (IECs). IECs and resident immune cells communicate with one another to reinforce barrier integrity, maintain homeostasis and mount robust responses against invading threats—schistosome eggs included. However, despite the host response against them, a large proportion of eggs successfully transit across the intestinal tissues and exit the host body. Crucially, to enable this process while limiting enteric inflammation, schistosomes have evolved several strategies to modulate host immune responses and manipulate host barriers. For example, as illustrated in Figure 2, schistosome eggs are capable of digesting through the intestinal muscular layer (muscularis mucosae) without triggering significant inflammation or immune infiltration. This process likely involves a collaboration between egg-derived proteases (yet to be defined) and immune cells that have been recruited to the serosal tissue and/or mesenteric vessels (24). In particular, the strategies employed by schistosomes to favor migration of eggs through the intestine likely heavily involve Th2 polarization and granuloma maintenance.

Moreover, due to infection longevity and the continuous passing of eggs, it is currently unclear, and difficult to assess, which intestinal layer schistosome eggs are predominantly located (i.e., serosa, submucosa, or mucosa). However, H&E images from infected mouse intestine show that eggs tend to penetrate through the muscularis-serosa layer en route to the lamina propria (64).

### Promoting Granuloma Formation

*S. mansoni* eggs are highly antigenic structures that continuously secrete a variety of innocuous, toxic or antigenic substances into host tissues (6). Accordingly, infiltrating eggs are focal points for the host immune system, which mounts a distinct attack and sequestration strategy in response: the granuloma. Granulomas are highly organized, multi-cellular structures that are enriched with a range of immune cells including Th2 cells, macrophages and eosinophils, with mast cell infiltration, accumulation of type 2 cytokines (such as IL-4, IL-13 and IL-5) and additional involvement from stromal cells/fibroblasts. Type-2 cytokines instruct the polarization of macrophages toward an alternatively activated (AA) phenotype, whom are essential to the resolution of egg-induced inflammation and tissue damage (65–67). After reaching maximal responses, granulomatous lesions decline in size and become fibrotic, with IL-13 being the main cytokine responsible for fibrosis, mediating its effects through AA macrophages and fibroblasts (5, 68, 69).

It is important to highlight that although these egg-triggered inflammatory reactions can also be found throughout infected liver, there are distinct differences between intestinal and hepatic granulomas in terms of size, cellular composition and extracellular matrix deposition (7, 70). These finding arguably reflect the higher proportion of dead eggs within infected liver, and so differences in egg secretions between the two tissues. Alternatively, they may suggest that the local tissue environment has a large bearing on granuloma function and development. In the case of intestinal granulomas, it would be very interesting to disentangle intestinal bacterial involvement, as studies on antibiotic-treated mice show only intestinal granulomas, but not hepatic granulomas, to be influenced by antibiotic administration (71). Moreover, it is unknown whether intestinal granulomas are better “designed” for egg excretion than their hepatic counterparts. Furthermore, while the immunology of hepatic granulomas has been studied extensively over past decades, our understanding of intestinal granulomas is considerably less. Accordingly, most of the data discussed in the following section has been obtained from studies on hepatic granulomas, and we cannot rule out the possibility that intestinal granuloma development may differ from their hepatic counterparts.

The schistosome granuloma is both beneficial and detrimental for the host (5). On the one hand, granulomas protect host tissues from egg-derived toxins such as Omega-1 and IPSE/alpha-1, which can cause severe damage (72). On the other hand, the fibrotic sequelae that follows granuloma resolution is the main cause of pathology and lethality in schistosomiasis (5). At the same time, in a remarkable example of co-evolution, granulomas are thought to be essential for schistosomes and continuation of their life cycle. Early studies in immunodeficient mice demonstrated the crucial role of granulomas in egg passage, with egg excretion rates plummeting in mice failing to generate intact granulomas, leading to lethal microvascular damage (11–14). These data also underscored the importance of host T cells in granuloma development, results that were later corroborated by studies in HIV^+^ infected people, in whom egg excretion rates were found to positively correlate with circulating CD4^+^ T cell levels (73). Furthermore, Th2 immunity is critical for intact granuloma development and host survival, since mice deficient in IL-4 and IL-13 signaling die during acute disease due to elevated intestinal and hepatic pathology, oxidative damage, endotoxemia and cachexia (65, 68, 74–76). Therefore, it comes as no surprise that schistosomes actively modulate host immune responses to ensure a Th2 bias is achieved.

Infection with *S.mansoni* follows a predictable immunological pattern, with a mixed, low level Th1/Th2 profile prevailing in the initial 4–5 weeks, then Th2 responses dominating from the point of egg production, with parallel expansion of regulatory cell networks that exert their influence most dramatically during chronic infection (77). Although many innate cell types help establish the Th2 milieu, dendritic cells (DCs) are essential (78) (79). Using their vast array of pattern-recognition receptors (PRRs), DCs sense, process and present schistosome egg-derived antigens to naïve CD4+ T cells, instructing Th2 development by yet to be fully defined mechanism (80).

SEA is a complex mixture of immune-stimulatory antigens, that is well known for its capacity to condition DCs for Th2 priming (81–84). Omega-1, a glycosylated T2 RNase, is a single component of SEA and potentially, the most powerful inducer of Th2 responses (85, 86). Omega-1 is taken up through mannose receptors on DCs and enhances Th2 polarization by degrading mRNA and ribosomal RNA, leading to reduced co-stimulatory molecules expression (CD83 and CD86) and reduced production of the Th1 promoting cytokine IL-12 (85, 86). Somewhat unexpectedly, SEA and Omega-1 can also stimulate DC production of Type-I IFN (normally associated with anti-viral responses), which enables their initiation of Th2 responses (87, 88). However, it is important to note that Omega-1 is not the only SEA component involved in DC conditioning, as SEA depleted of Omega-1 and *S.mansoni* eggs with specific Omega-1 knock-down still retain some of their Th2 polarizing potential (85, 89). In addition, Kaisar and colleagues very recently identified a novel pathway in Th2 polarization that entails Dectin1/2 signaling on DCs and works independently of the actions of Omega-1 (90). Overall, the impact of egg antigens on host innate immune cells is complex and multifactorial, often flouting convention, but with the cumulative effect being initiation of a strong Th2 response.

It is important to realize that although Th2 skewing is primarily ascribed to eggs, adult worms are also capable of instructing a Th2 environment before the onset of egg production (91). In fact, by comparing granuloma formation between models of natural infection and portal vein egg injection, Leptak and colleagues demonstrate that granuloma development requires both worm- and egg-derived signals (92). Notably, while the inflammatory reaction toward portal vein injected eggs is minimal in terms of reaction volume, cellular infiltration and collagen deposition, these reactions can be sufficiently restored if eggs are injected into mice previously infected with single-sex male cercariae or exposed to adult worm homogenates (92). Mechanistically, this ‘priming' effect could be explained by the sharing of antigens between adult worms and eggs (92). Further, cytokines produced in response to adult worms may be key to granuloma induction (23, 42, 92). In particular, the cytokine TNF is thought to be exclusively produced in response to adult worms, and may be able to restore granuloma formation in naïve mice injected with eggs alone (92). Interestingly, it has been suggested that the production of TNF may be linked to phagocytosis of worm-regurgitation products by local macrophage populations (92, 93).

The schistosome granuloma is hallmarked by the accumulation of AA macrophages, which predominantly arise from the recruitment of Ly6C^Hi^ monocytes, as opposed to the proliferation of local macrophage populations (94, 95). AA macrophages are activated by type-2 cytokines (IL-4/IL-13), characterized by the expression of various signature genes (such as arginase 1, Ym1, Relmα) and are thought to prevent potentially lethal host pathology via a variety of mechanisms (67, 96). This includes coordinating the recruitment of collagen and cells to developing granulomas, regulating T-cell proliferation, facilitating tissue repair and regeneration, and inhibiting the differentiation of classically activated macrophages (CAMs), which generate pro-inflammatory cytokines and cause oxidative tissue damage (67, 97, 98). Crucially, thanks to the wound healing capacities of AA macrophages, intestinal barrier integrity is sufficiently maintained during egg migration and the host is shielded from enteric bacteria (67). Indeed, in mice deficient in IL-4/IL-13 signaling specifically on macrophages and neutrophils [LysM(Cre)IL-4Ralpha(-/flox) mice], AA macrophages do not develop and mice are extremely susceptible to *S. mansoni* infection (100% mortality during acute disease) (65). Importantly, acute mortality was associated with exaggerated Th1 pathology, severe hepatic and intestinal damage, impaired egg excretion and sepsis (65). In more recent studies, AA macrophages have also shown to maintain tissue integrity in models of urogenital schistosomiasis (66), and their protective function has been partially attributed to the enzyme arginase-1 and resistin-like molecule (RELM)-α (98–101). Notably, arginase-1 contributes to the long-term survival of schistosomiasis by restricting Th1 and Th17 associated immunopathology, and RELM-α acts as a negative regulator of Th2 responses (98–101). Moreover, while YM-1 is an additional phenotypic marker of AA macrophages, its contribution to Th2 responses during schistosomiasis remains undefined. However, various reports suggest a role for YM-1 in Th2 differentiation (102, 103). Of note, our understanding of the complexity of ‘M2' macrophages, and the range of cytokines that can promote aspects of M2 polarization, is continually growing, with IL-10, TGF-β and IL-33 also shown capable of instructing alternative activation (61, 104–106).

Schistosomes not only rely on cytokines to instruct AA macrophages induction, but also play a direct role in their polarization. Schistosome-derived molecules that may contribute to alternative activation include SEA, peroxiredoxin, lysophosphatidylcholine and hemozoin (101, 107–110). SEA, for instance, appears able to directly interact with macrophages to instruct an AA phenotype, or indirectly elicits an AA profile via IL-33 and its receptor ST2 (61, 109). Interestingly, in a model of intravenous *S.mansoni* egg injection, mice deficient in ST2 demonstrated impaired production of Th2 cytokines and primary granuloma development (111). However, this study did not assess the influence of ST2 deficiency on macrophage function or polarization. Another molecule that influences macrophage polarization is hemozoin. Hemozoin is a neutralized version of heme that schistosomes regurgitate into the bloodstream following the digestion of host erythrocytes (110). Hemozoin crystals spontaneously aggregate in the liver, where their consumption by patrolling macrophages promotes an alternatively activated phenotype, including the expression of the Th2 negative regulator RELM-α (99–101). Moreover, the uptake of these worm-regurgitation products by antigen-presenting cells has been suggested to induce the production TNF, which has previously shown a key role in granuloma formation (23, 42, 92).

In terms of other myeloid cells, eosinophils and mast cells are cardinal features of the schistosome granuloma, but their function there remains elusive. While older studies suggest that eosinophils favor egg passage by digesting the epithelial basal membrane, more recent models of eosinophil ablation found no obvious role for them in this process or for granuloma formation, fibrosis or worm burden (112–114). In this context, mast cell function also remains a mystery. During *Trichinella spiralis* infections, mast cells mediate parasite expulsion and disrupt epithelial barrier function through the release of mast cell protease 1 (mMCP-1), a serine protease that degrades TJ proteins (115). These observations prompted investigations into the role of mMCP-1 in barrier integrity during schistosome infections, but no functional role was found in this setting (116). However, mast cell secretion of pro-angiogenic factors could support egg extravasation (117). Additionally, the strong Th2 milieu may simply promote the recruitment of eosinophils and mast cells to the area, where it is possible that both cells types represent (minor) innate sources of Th2 associated cytokines, and so help sustain granulomas.

The alarmins IL-33, IL-25 and TSLP (thymic stromal lymphopoietin) are important initiators of type 2 immune reactions that are released from the epithelium, endothelium and other stromal compartments upon damage and stress (118). Although yet to be formally demonstrated, their release could be triggered by egg migration. While individual ablation of TSLP, IL-25, or IL-33 has no discernible impact on hepatic granuloma formation and fibrosis during chronic *S. mansoni* infection, simultaneous blockade of all three signaling pathways results in a modest decrease in Th2 associated pathology by 9 weeks of infection, including a reduction in granuloma-associated eosinophilia, hepatic fibrosis and IL-13 producing type 2 innate lymphoid cells (ILC2s) (119, 120). However, such effects were transient in nature and no longer significant by 12 weeks post infection, suggesting that these alarmins are dispensable for the development and maintenance of egg-induced pathology (120). Similarly, the observed reduction in ILC2 activity appeared to be compensated for by enhanced CD4+ T cell responses, indicating that ILC2s also have no major function in the maintenance of type 2 inflammation in this particular setting. Considering the essential role of Th2 responses in granuloma formation and egg expulsion, it makes sense that compensatory mechanisms are at play, and that schistosomes are not overly reliant on the unregulated release of stromal mediators for development or type 2 immunity. However, given that ILC2s have been shown to be important initiators of Th2 responses during gastrointestinal helminth infections, and to significantly contribute to wound repair responses at mucosal surfaces, further studies are required to reach a formal conclusion on the involvement of ILC2s in schistosome-associated Th2 inflammation (121–123). In addition, although the aforementioned study signposts the redundancy of alarmins in type 2 immunity during schistosome infection, there remains the possibility that they contribute to regulatory cell expansion during chronic stages of disease (124).

### Exiting the Epithelium

Tight junctions (TJs) are the final hurdle that schistosome eggs must overcome to transit through the intestinal wall and exit the host. TJs are multi-protein complexes that tightly link adjacent IECs at their apical membranes, creating virtually impermeable seals. In this manner, TJs regulate para-cellular permeability (the movement of substances between adjacent cells) and are essential for maintenance of intestinal barrier function (125). In scenarios of TJ disruption, intestinal barrier function is impaired, allowing for enhanced permeation of luminal substances (such as bacteria, antigens, toxins and metabolites) into mucosal tissues and the systemic circulation (125). Both experimental models and human infection studies hint toward increased intestinal permeability during schistosome infections. Mice experimentally infected with *S. mansoni* demonstrate reduced ileal integrity from 8 weeks post infection, and *S. mansoni* infections in humans are associated with extremely high levels of endotoxin in the bloodstream (116, 126). In addition, the lethality of experimental schistosomiasis in scenarios of AA macrophage impairment is partially attributed to mass disruption of the intestinal wall and sepsis, with a common gastrointestinal symptom of intestinal schistosomiasis being blood in stool (65, 127).

One future goal is to better define schistosome-epithelial interactions. Namely, is intestinal epithelial disruption solely driven by damage caused by egg migration and/or, do schistosomes actively modulate components of the epithelial barrier to favor egg migration? In a unique model of urogenital schistosomiasis, in which *S. haematobium* eggs are directly introduced to the bladder wall, eggs were shown to suppress the transcription of multiple genes implicated in urothelial barrier maintenance, including junctional adhesion molecules claudin and uroplakin (128). These data indicate that schistosome eggs not only physically disrupt TJs as they pierce through the intestinal lining, but also suppress the transcription of various TJ related genes, which could facilitate egg transit. However, in a separate study, *S. japonicum* eggs were instead found to reinforce intestinal epithelial barrier function and relieve inflammation in the TNBS colitis model (129). Crucially, prevention of experimental colitis was accompanied by reduced bacterial translocation and enhanced levels of tight junction molecules (Zo-1 and occludin). This study suggests that eggs can fortify the intestinal barricade to prevent potentially lethal bacterial translocation, and thereby enhance host survival. Perhaps the discrepancies between these two studies reflect the manner of egg introduction: direct egg injection into the bladder wall vs. injection into the peritoneal cavity. More specifically, could the introduction of eggs into the peritoneal cavity influence the function of intestinal cell types? Direct injection of eggs into intestinal tissue, as very recently described (130), may provide a novel approach to study the impact of *S. mansoni* eggs on intestinal barrier integrity.

In addition to direct effects of schistosome eggs, intestinal permeability can be influenced by cytokines such as IFN-y, TNF and IL-13, as well as epithelial cell apoptosis and exogenous factors such as diet and non-steroidal anti-inflammatory drugs (125, 131). With regards to cytokine-mediated barrier dysfunction, it was recently demonstrated that schistosome eggs are incapable of penetrating through epithelial cells themselves, but require pro-inflammatory mediators TNF and/or IFN-γ to disrupt tight junctions and reduce intestinal epithelial resistance (71). Schistosome eggs appear not to stimulate production of pro-inflammatory cytokines by DCs, which suggests that other local mediators or cell types may be involved (71, 80). Alternatively, the tissue damage caused by egg transit, compounded by involvement of intestinal bacteria, could conceivably stimulate production of such cytokines (71). Furthermore, IL-13 has shown to induce epithelial apoptosis and increase the expression of the pore forming TJ molecule claudin-2, indicating that Th2 responses themselves can be instrumental to decreased barrier integrity (131)_._

Once eggs rupture past IECs they are met by a stratified layer of mucus, whose nature and function in schistosomiasis remains undefined. Mucus secretion is likely enhanced during schistosome infections, given that the IL-4/IL-13 signaling axis is critical to goblet-cell hyperplasia and mucus secretion (132, 133). Whether such alterations influence egg expulsion is unclear. In gastrointestinal nematode infections, mucus mediates the rapid expulsion of these parasites by limiting their motility and preventing their establishment within the gastrointestinal tract (132). Applying these observations to schistosome infections, it is possible that increased mucus generation leads to accelerated expulsion of eggs into the environment. In addition, since intestinal mucins are capable of instructing important pro-inflammatory functions in DCs, it is tempting to suggest that mucins may contribute to modulation of the inflammatory environment during schistosomiasis (134). Directly related to this, the exposure of DCs to mucins may be increased by egg-induced disruption of the intestinal barrier.

While larval and adult gastrointestinal nematodes likely interact with IECs for prolonged periods of time, schistosome eggs transiently move past or through them during transit. It is unknown whether these different exposure times influence how IECs sense and respond to the parasite infections. Very recently, intestinal tuft cells (a rare IEC population) were shown to instigate type 2 immunity in response to gastrontestinal nematodes infections (135–138). As tuft cell research is still in its infancy, defining the interactions between tuft cells and schistosome eggs remains an open and interesting point of further study.

## Microbial Mediated Migration

In the face of the complexity of the immunopathology they generate, schistosome eggs ultimately transit from the mesenteric vessels via the serosa, the muscularis, the epithelium and the mucosa into the intestinal lumen, where a dense and vibrant microbiota surrounds them. As discussed above, successful egg penetration requires carefully coordinated interactions between host and parasite. These interactions can be extended to a third partner: the intestinal microbiome (herein, defined as commensal bacteria, viruses and fungi).

Helminths and the microbiome have co-evolved with their mammalian hosts over millennia, so extensive interactions exist between the three parties (139). Many helminths favor the establishment of defined microbial communities to support their own infectious life cycle and improve the overall wellbeing of the host. For instance, *Trichuris muris* uses intestinal bacteria as environmental hatching cues and *Heligmosomoides polygyrus* infections alter cecal microbiome composition, leading to a greater availability of short chain fatty acids (SCFAs) that dampen allergic responses (140, 141). While the interface between host, microbiota and intestinal-dwelling nematodes is beginning to be understood, there is currently a paucity of information on this topic about schistosomes, and whether they also remodel gut microbiome composition to support their own life cycle and lessen host pathology.

In mice, experimental *S. mansoni* infections are accompanied by profound changes in microbiota composition from at the point of egg production, including the expansion of *Akkermansia muciniphila* and bacterial populations from the Family Lactobacillaceae (142). The expansion of these bacterial communities may favor chronic schistosome infection by elevating the frequency of regulatory cell populations, or repairing egg-induced damage to the intestinal wall (143, 144). Further, a recent study using a broad spectrum of antibiotics showed that bacterial depletion reduces fecal egg excretion and intestinal granuloma development during murine infection with *S. mansoni* (71). In contrast, worm fecundity and liver pathology was not influenced by antibiotic administration, indicating that intestinal granuloma development and egg transit is particularly dependent on local, bacterially-derived factors. These factors could include bacterially-induced cytokines, such as IFN-y and TNF, that are needed for tight junction severance (71, 125). Complementing these studies, metabolic analysis of feces from mice experimentally infected with *S. mansoni* reveals several alterations in gut-bacteria related metabolites from day 41 of infection, including a greater availability of the SCFA, propionate (145). Additionally, differences in intestinal microbiome structure have been reported between children with or without *S. haematobium* infection (146). As *S. haematobium* is a urogenital parasite, this observation indicates that the schistosomes have a systemic influence on microbiome composition. Similar to that observed in *Trichuris* infection, it is possible that the schistosome-induced Th2 responses promote the establishment of a particular microbiome (147). Moreover, as signals from the gut microbiome are known to influence immune responses at both local and distal locations (e.g., the lung), it is possible that schistosome-induced alterations to the intestinal microbiome influence the development and/or protection against a range of diseases (148, 149).

## The Immune Implications of Egg Penetration

In mice, granulomatous responses are maximal by approximately 8–10 weeks post infection, before gradually declining in magnitude (150, 151). This decline corresponds with a reduction in lymphocyte proliferation and responsiveness, and represents the transition from acute to chronic disease (77, 152, 153). Chronic schistosomiasis is characterized by high circulating levels of anti-inflammatory IL-10 and/or TGF-β and the profound expansion of regulatory T cells (Tregs) and B cells (Bregs), which function to supress potentially deleterious activities of T-helper cells and limit granulomatous pathology (77). Schistosome eggs are continually produced during this chronic period, but thanks to the immune-modulatory capacities of these regulatory cell networks, the majority of chronically infected individuals do not develop lethal pathology.

Schistosomes drive regulatory cell induction via a variety of immunomodulatory influences (154). The egg-derived glycoprotein IPSE-1/ alpha-1,was recently shown to promote B cell IL-10 and equip B cells with Treg inducing capacities (155). In the case of worm-derived products, both Cyclophilin A and schistosomal lysophosphatidylserine (lyso-PS) can modulate DC function, leading to preferential expansion of IL-10 producing Tregs (156, 157). Small exosome-like extracellular vehicles (EVs) from schistosomes could also represent untapped and important sources of immunomodulation (158, 159). In addition to such direct effects of worm and egg-derived products on inflammation and the immunological environment, it is tempting to speculate that egg penetration itself will shape the immunoregulatory landscape, perhaps in part via facilitating the systemic spread of immune-modulating luminal molecules.

Such molecules could include LPS and bacterially-derived metabolites. SCFAs, for instance, are known to increase Treg responses, mediate the dampening of allergic airway inflammation in the context of *H. polygyrus bakeri* infection, and down-regulate the pro-inflammatory effector functions of intestinal macrophages (140, 149, 160, 161). A role for SCFAs in schistosome-driven immune regulation has not yet been demonstrated, but this would make for an interesting point of further study. Intriguingly, although reported endotoxemia levels are 10 times higher in sera from individuals with human schistosomiasis than that observed in lethal toxic shock, study participants have neither poor health nor systemic inflammation (126). This observation has been suggested to reflect the type of LPS in the blood stream, where the structure of LPS (specifically Lipid A acylation) dictates whether it has an antagonistic or agonistic effect (126, 162). As the structure of LPS varies between different bacterial communities, potential differences in LPS immunogenicity could reflect schistosome-induced alterations to the gut microbiome (142, 162). Alternatively, it is possible that schistosomes modulate innate cell responsiveness to TLR ligands and/or reprogram innate cell function to acquire a more regulatory phenotype (71, 156, 163, 164). In support of these hypotheses, schistosome-derived lysophosphatidylserine has been shown to be capable of modifying TLR2 signaling pathways in DCs, leading to altered maturation and enhanced induction of Tregs (156). In addition, *S. japonicum* infections can increase the survival rate of mice with LPS-induced sepsis, in a mechanism that likely entails the polarization of macrophages to an M2 phenotype (165, 166). Molecules that may mediate this effect include schistosome-derived cysteine proteases, which were recently shown to protect against sepsis challenge and lower the production of pro-inflammatory cytokines and NO from LPS-stimulated macrophages (167). Interestingly, similar protective mechanisms appear to be induced during infections with the filarial worm *Litomosoides sigmodontis (*163*)* and the trematode *Fasciola hepatica* (168). Notably, *F. hepatica* reduces the release of inflammatory mediators from macrophages via mechanisms of molecular mimicry, and *L. sigmodontis* protects from bacterial sepsis by down-regulating the expression of macrophages genes involved in TLR signaling (163, 168).

The translocation of bacterial ligands into host tissues likely has immunomodulatory consequences. In the absence of Myd88 signaling, which is central to bacterial-innate cell interactions, schistosome infected mice demonstrate impaired Th1 responses and reduced granulomatous responses (169). The regulatory environment may also be influenced by microbial translocation, where translocated LPS could combine with SEA to trigger the release of inflammasome-derived IL-1β, which partakes in Breg induction (170, 171). Finally, there remains the possibility that bacterial leakage influences the dynamics of macrophage proliferation and alternative activation at the site of inflammation. For example, during experimental schistosomiasis, it is possible that intestinal bacteria act as a trigger for monocyte recruitment from the bone marrow and resultant macrophage accumulation, as opposed to the *in situ* proliferation of resident macrophage populations (95, 172).

By strategically expanding regulatory cell populations, schistosomes not only limit egg-induced pathology, but can also suppress the development of various inflammatory diseases including allergy (173). Indeed, many epidemiological studies have found inverse correlations between schistosomiasis and allergies, and in experimental models schistosome infection provides relief against allergic airway inflammation and OVA-induced airway hyper-responsiveness, with optimal protection achieved during chronic but not acute disease stages (174–178). There is also evidence to show that schistosome eggs can protect from allergic asthma in the absence of adult worms, despite the strong Th2 responses they evoke (179, 180). A current research goal is to better define the immunomodulatory mechanisms employed by schistosomes, where this new-found knowledge could potentially be used to reengineer and recover regulatory cell function in allergic individuals.

## Eggs that Fail

Despite the multiple strategies employed, many eggs fail to exit their mammalian host. Experimental infections indicate that only 20–55% of eggs are successful excreted, while the remainder inevitably become trapped within host tissues (3, 4). Although egg deposition is targeted for the urinary bladder (*S. haematobium*) and the intestine (S*. mansoni* and *S. japonicum*), eggs swept systemically through the blood stream can readily be detected at various other locations including the eyes, skin, kidney, spleen and central nervous system (CNS) (4, 16). Egg deposition at intended and unintended sites can have serious pathological consequences for the host. The more severe disease complications generally manifest many years after infection, reflecting gradual egg accumulation in host tissues and the resolution of granulomas by fibrosis and calcification (127).

This aspect of schistosome infection is perhaps most evident in liver pathology during *S. mansoni* and *S. japonicum* infection. Granuloma formation around hepatic egg deposits leads to severe fibrosis, blood flow obstruction and, subsequently, the formation of ascites and blood vessels that bypass the liver (portosystemic shunting) (127). If these vessels rupture, life-threatening bleeding may follow. Pathology in the intestine is generally less severe than in the liver, and can be characterized by pseudopolyposis, ulceration and stricture formation. In *S. haematobium* infections, egg entrapment within pelvic organs (urinary bladder, ureters, cervix, vagina, prostate gland and seminal vesicles) can also result in severe pathology, including obstructive bladder and ureteral fibrosis, bladder cancer, kidney failure and the formation of gross genital sores (127, 181). In females, these lesions may give rise to infertility, ectopic pregnancies and menstrual irregularities, while in men, higher rates of sperm apoptosis have been reported (181, 182).

Importantly, reduced integrity of the genital epithelium constitutes a significant risk factor for the acquisition of HIV and other pathogens, including oncogenic viruses and bacteria (183, 184). Indeed, a recent longitudinal study in Tanzania demonstrated a clear gender bias in schistosomiasis and HIV acquisition, with schistosome-infected women demonstrating a 3 fold higher chance of acquiring HIV than uninfected women, whereas odds remained the same for men with or without infection (185). The increased risk of HIV acquisition in females very likely reflects the local physical changes caused by schistosome eggs at the female genitalia (183). The sequestration of eggs within the mucosal tissue of the vagina and cervix leads to ulceration, erosion and the formation of tiny cervical abnormalities (yellow and/or grainy sandy patches) that are surrounded by an irregular network of blood vessels. These blood vessels are believed to represent egg-induced angiogenesis and could accelerate the transmission of HIV during intercourse (52, 183). In contrast, *S. haematobium* infected men are at lesser risk of acquiring HIV because eggs do not infiltrate male genital organs that are exposed to the virus. However, other studies have shown mild associations between male urogenital schistosomiasis and HIV-1 acquisition, and even though *S. mansoni* eggs do not localize to the genitalia, *S. mansoni*-infected individuals also appear more susceptible to HIV (183, 186, 187). These associations are believed to be a consequence of schistosome-associated immunomodulation, as opposed to local tissue damage caused by eggs (183). In particular, the co-receptors for HIV are more highly expressed on the surface of CD4+ T cells from schistosome-infected individuals, allowing for greater viral uptake (186).

Similar to HIV, there is a clear link between urogenital schistosomiasis and bladder cancer, with promotion of carcinogenesis likely due to *S. haematobium* enhancing entry sites for oncolytic viruses and bacteria (184, 188). Alternatively, egg-driven inflammation may have a bystander effect on host cells, or immunosuppressive cytokines (produced during chronic stages) may reduce host ability to clear oncolytic viral and bacterial infections. In addition, *S. haematobium* eggs have shown to directly influence the transcription of carcinogenesis-associated genes within the bladder wall (128). This effect could potentially be mediated by schistosome-derived estrogenic molecules, albeit their carcinogenic activity has yet to be defined (189).

Curiously, while a clear association between *S. haematobium* and bladder cancer has been established, there is limited and controversial evidence to implicate S. *japonicum* and S*. mansoni* in intestinal and liver cancer (184, 188, 190). The reason for these strain-specific differences is unclear, but it has been suggested this reflects the site of egg deposition and/or the contribution of environmental carcinogens, including tobacco smoke or industrial and agricultural dyes (184, 188).

The spreading of eggs to the CNS (neuroschistosomaisis) is one of the more devastating outcomes of schistosome infection that can result in seizure or paralysis, depending on brain or spinal cord involvement. While *S. japonicum* eggs are more frequently found in the brain, eggs from *S. mansoni* and *S. haematobium* appear to have a predilection toward the spinal cord (127). These differences could reflect the smaller size of *S. japonicum* eggs and/or their absence of a protruding spine (127). Moreover, given that schistosome eggs disperse to many ectopic locations, it is possible that they affect co-infections encountered at these sites too. At a local and mechanistic level, the inflammatory environment established by eggs may exacerbate already established pathology or create an environment that favors other pathogen survival. At an immunological level, schistosome co-infections may suppress immune responses toward viral and bacterial antigens, leading to ineffective pathogen clearance and chronicity (191, 192).

## Conclusion and Outlook

How schistosome eggs successfully exit the host body has long been a focus of many researchers. Very early studies revealed the severity of egg-driven pathogenesis and the curious migration patterns taken by adult worms to reach favored sites of oviposition (3, 16, 17, 51, 181). In more recent years, research has shed light on the molecular and immunological mechanisms that govern this process. Within host blood vessels, schistosomes establish a suitable environment for maturation, movement and egg extravasation by interfering with host haemostasis and angiogenesis (9, 10). For intestinal egg passage and granuloma formation, schistosomes actively tamper with host immune responses, to achieve a delicate balance between immune effector and immune regulatory activity, and to limit bystander tissue damage. However, despite the exit strategies employed, egg transit is not a certainty and lethal pathology may follow egg entrapment.

Our understanding of the interactions between schistosomes and the mammalian host is continually growing, with many interesting avenues still open for exploration (see Box 1). For example, while schistosome infection influences gut microbiome composition, we are yet to define how these fluctuations impact egg migration, disease pathogenesis, immunomodulation and long-term host/parasite survival (142). With powerful sequencing techniques emerging and our knowledge of the intestinal microbiome constantly expanding, we anticipate that the relationship between the mammalian host, schistosomes and the microbiome will soon be much better understood. Moreover, we are optimistic that the study of schistosomes will continue to increase fundamental understanding of the mechanisms governing immune-regulation and type 2 immunity. In turn, this could lead to the identification and generation of new vaccine candidates and targets for the treatment of egg-induced pathogenesis in schistosomiasis, as well as other type 2 inflammatory diseases.

Box 1 **Outstanding questions**

- Recent studies show schistosome infections to influence the composition of the gut microbiota. Do these changes reflect bacterial adaptations to the inflammatory environment established by schistosomes infections, or do schistosomes actively promote the colonization of select bacterial communities? Furthermore, do these microbiome fluctuations influence (i) egg excretion, (ii) pathogenesis, (iii) the immune landscape, and/or (iv) schistosome long-term survival?
- Which exact molecules mediate egg binding to the vascular endothelium, and which process(s) ultimately allow for egg extravasation?
- The effects of schistosome infections on cancer require further attention. Notably, why is the association between carcinogenesis and schistosome infection stronger for S. *haematobium* than for S. *mansoni* and *S. japonicum*?
- Many cells of the intestinal immune system have undefined roles in the instigation and maintenance of type 2 immunity during schistosome infection. Notably, what is the actual function of eosinophils and mast cells in the granuloma? Are chemosensory tuft cells involved in egg detection and the initiation of type 2 responses? Which intestinal macrophage subsets are involved? Do ILC2s participate in these immune reactions?
- Do worms and/or eggs secrete molecules that influence host intestinal barrier integrity? Does the secretion of such molecules promote egg penetration through host tissues?
- Which co-infections/pathologies in the gut are worsened by the inflammatory environment established by eggs?
- Does egg migration trigger the release of alarmins (e.g., IL-25, IL-33, and TSLP) in the intestine, and do these molecules influence the development of Th2 or regulatory responses in the gut?
- Serosal immune responses are poorly characterized. Do serosa-resident immune populations play a role in egg migration and how do eggs transit through these tissues?
- Mucins - Do they play a functional role in intestinal egg-migration or local immune modulation

## Author Contributions

AC wrote the manuscript, AM and HS critically reviewed and modified the manuscript.

### Conflict of Interest Statement

The authors declare that the research was conducted in the absence of any commercial or financial relationships that could be construed as a potential conflict of interest.
