# Corrigendum: Schistosome Egg Migration: Mechanisms, Pathogenesis and Host Immune Responses

**DOI:** 10.3389/fimmu.2019.00749

**Published:** 2019-04-11

**Authors:** Alice H. Costain, Andrew S. MacDonald, Hermelijn H. Smits

**Affiliations:** ^1^Department of Parasitology, Leiden University Medical Center, Leiden, Netherlands; ^2^Lydia Becker Institute of Immunology and Inflammation, University of Manchester, Manchester, United Kingdom

**Keywords:** *Schistosoma mansoni*, intestine, endothelium, type 2 immunity, immune modulation

In the original article, we neglected to include the funders the “MRC, MR/N013751/1” and the “BBSRC, BB/P504543/1” to AM.

The authors apologize for this error and state that this does not change the scientific conclusions of the article in any way. The original article has been updated.

